# Longitudinal Trajectories in Essential Tremor: Evidence From A Seven‐Year Follow‐Up of Motor and Non‐Motor Symptoms

**DOI:** 10.1111/ene.70646

**Published:** 2026-06-01

**Authors:** Luca Angelini, Daniele Birreci, Anna Sofia Grandolfo, Martina De Riggi, Davide Costa, Adriana Martini, Sara Cirinei, Simone Aloisio, Giulia Paparella, Rick C. G. Helmich, Matteo Bologna

**Affiliations:** ^1^ IRCCS Neuromed Pozzilli Italy; ^2^ Department of Human Neurosciences Sapienza University of Rome Rome Italy; ^3^ Department of Neurology and Stroke Unit San Camillo de Lellis, General District Hospital Rieti Italy; ^4^ Department of Translational and Precision Medicine Sapienza University of Rome Rome Italy; ^5^ Neurophysiopathology Unit, Department of Translational Biomedicine and Neuroscience University of Bari Aldo Moro Bari Italy; ^6^ Centre for Cognitive Neuroimaging Donders Institute for Brain, Cognition and Behaviour, Radboud University Nijmegen Nijmegen the Netherlands; ^7^ Department of Neurology, Center of Expertise for Parkinson and Movement Disorders Donders Institute for Brain, Cognition and Behaviour, Radboud University Medical Centre Nijmegen the Netherlands

**Keywords:** disease progression, essential tremor, essential tremor plus, kinematic analysis, longitudinal study

## Abstract

**Background:**

Essential tremor (ET) is a common movement disorder characterized by heterogeneous features, though the rate and pattern of worsening are variable. Longitudinal data combining clinical and objective motor measures remain limited, resulting in uncertainty regarding trajectories and predictors of progression in ET.

**Methods:**

Twenty‐two patients from a previously established ET/ET‐plus cohort underwent a third clinical and kinematic evaluation almost seven years after baseline. Kinematic analysis assessed postural, kinetic, rest tremor, and finger‐tapping performance. Longitudinal changes were evaluated using non‐parametric statistics and linear mixed‐effects models adjusting for clinical covariates.

**Results:**

Over time, tremor worsened overall. Kinematic measures indicated a selective increase in kinetic tremor severity, while rest and postural tremor amplitude remained stable. Clinically, tremor spread to a greater number of body regions. Soft neurological signs increased over time and were associated with greater tremor spread. Cognitive performance showed only a mild decline, partly related to age and affective symptoms. Higher baseline tremor severity was associated with greater worsening of kinetic tremor, and lower baseline cognitive scores with changes in postural tremor.

**Conclusions:**

ET progression is heterogeneous and often non‐linear. Kinematic measures were particularly sensitive in capturing worsening of kinetic tremor, the most disabling clinical feature. The accumulation of soft neurological signs and their association with tremor spread support the notion of ET‐plus as a more advanced disease stage with broader cerebral involvement. Independent of disease progression, there were strong aging‐related effects. Future multimodal longitudinal studies may clarify how aging interacts with disease trajectories in essential tremor.

## Graphical abstract

Long‐term follow‐up (almost 7 years) of an ET/ET‐plus cohort (*n* = 22) shows heterogeneous and partly non‐linear progression, with a contribution of aging to disease burden. Tremor spreads anatomically over time, with selective worsening of kinetic tremor and increasing burden of soft neurological signs. Quantitative kinematic assessment complements clinical evaluation, enabling sensitive characterization of disease trajectories.

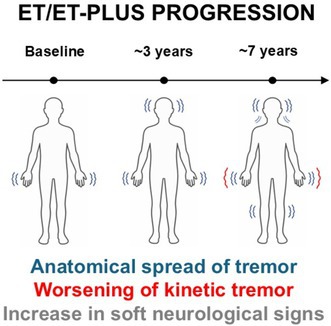

## 1 Introduction

Essential tremor (ET) is the most common movement disorder, with prevalence increasing with age [1], and is defined by a bilateral action tremor of the upper limbs lasting at least three years, in the absence of other neurological signs [2]. However, a substantial proportion of patients also exhibit additional neurological features of uncertain significance, known as ‘soft signs’, which may include subtle parkinsonism (e.g., rest tremor and bradykinesia) or dystonia, and are therefore classified as ET‐plus [3, 4]. The heterogeneous clinical phenomenology of ET reflects its complex pathophysiology, which is thought to involve dysfunction of the cerebello–thalamo–cortical circuitry [5]. However, additional areas, including the basal ganglia and frontal lobe circuits, may contribute to ET pathophysiology [6, 7, 8].

Longitudinal studies indicate that ET is a progressive condition [9], though the rate and pattern of worsening are variable and often non‐linear [10, 11]. Existing longitudinal studies are limited in number and constrained by small sample sizes or the absence of objective motor assessments, resulting in uncertainty regarding predictors of progression. In our previous longitudinal study, we observed that tremor severity increases over time, with spread from the upper limbs to other body regions [12]. Again, soft neurological signs become more frequent as ET progresses [12, 13, 14]. However, significant gaps remain in our understanding of the natural history of ET. It is still unclear how tremor severity along with soft signs, cognitive decline, and psychiatric symptoms evolve together, and whether they reflect distinct or converging pathophysiological mechanisms. Several factors have been proposed as predictors of greater tremor progression, including older age, later onset, female sex, baseline rest tremor, and impaired tandem gait. However, some of these findings remain inconsistent [12, 14, 15, 16, 17]. Furthermore, the longitudinal relationship between motor and cognitive trajectories, and the prognostic significance of soft signs, remain poorly understood.

The current work builds on a previous longitudinal cohort [12] and extends follow‐up in a subgroup of participants, thereby providing one of the longest prospective studies to date, combining longitudinal clinical motor phenotyping with quantitative movement analysis and an extended assessment of cognitive and affective domains. Our objectives were: (i) to characterize the progression of tremor severity, its anatomical distribution and the co‐occurrence of soft signs; (ii) to integrate objective kinematic data with clinical observations; and (iii) to explore baseline factors associated with longitudinal change.

## 2 Methods

### 2.1 Participants and Study Design

This study represents the third assessment in a previously published longitudinal cohort of 37 patients diagnosed with ET or ET‐plus, consecutively recruited at the Movement Disorders outpatient clinic of Sapienza University of Rome since 2018 [12]. All patients were initially diagnosed according to the consensus criteria of the International Parkinson and Movement Disorder Society [2] by a senior neurologist specialized in movement disorders. Inclusion and exclusion criteria were consistent across all time points. Patients were excluded if they had neurological disorders other than ET, cognitive or physical impairments affecting upper‐limb movement and preventing completion of the kinematic assessment, or a history of alcohol abuse or exposure to tremor‐inducing medications.

At each time point, patients underwent both a standardized clinical examination and a kinematic assessment using the same protocol previously described [12]. Assessments were performed at the same time of day, in the off‐therapy condition after standardized dose tapering and washout (24 h for propranolol and benzodiazepines; 48 h for primidone and topiramate) [12] and after abstinence from alcohol and stimulants for at least 24 h. Clinical assessments were performed by two neurologists specialized in movement disorders and video‐recorded for review by a senior specialist to achieve consensus in cases of discrepancies. Kinematic recordings were reanalyzed using a standardized procedure whenever differences were found in previous pipelines, as detailed below.

Twenty‐two patients from the original cohort of 37 completed the second follow‐up, representing 59.5% of the initial participants. Mean follow‐up intervals were 36.36 ± 11.30 and 42.27 ± 10.02 months, with a total follow‐up duration of 79.05 ± 13.25 months. Reasons for dropout included withdrawal of consent (26.7%), loss to follow‐up despite repeated contact attempts (26.7%), personal or social difficulties (13.3%), new diagnosis of dystonia (13.3%), new diagnosis of Parkinson's disease (PD) (6.7%), unrelated medical conditions (6.7%), and death (6.7%). Patients who received an alternative neurological diagnosis during follow‐up were not included in the third kinematic assessment and were therefore excluded from the present longitudinal analyses. This decision was made because maintaining diagnostic consistency across time points was considered essential to ensure the interpretability of within‐subject longitudinal trajectories. No significant differences in baseline demographic or clinical characteristics were found between participants who completed follow‐up and those who dropped out (Table SS1). The study was approved by the local Ethics Committee and conducted in accordance with the Declaration of Helsinki. All participants provided written informed consent.

### 2.2 Clinical Assessment

Motor severity was evaluated with the Fahn–Tolosa–Marin Tremor Rating Scale (FTM‐TRS) and the motor section of the Unified Parkinson's Disease Rating Scale (MDS‐UPDRS part III). Rest tremor, subtle bradykinesia, mild dystonic posturing, tandem gait difficulty, and MCI were considered soft signs, as described in a prior study [12], except for MCI, which was defined as a Montreal Cognitive Assessment (MoCA) score < 26 [18]. Psychiatric symptoms were quantified using the Hamilton Depression (HAM‐D) and Anxiety (HAM‐A) Rating Scales. Psychiatric scores were used only for correlation analyses and as covariates for linear mixed‐effects models (LMMs); no formal psychiatric diagnoses were assigned. None of the patients were receiving cognitive enhancers or antidepressants during the study. Dementia was defined based on clinical judgment, integrating cognitive impairment with functional decline in activities of daily living.

### 2.3 Kinematic Assessment

Quantitative motor assessment was performed using an optoelectronic motion capture system (SMART DX, BTS Bioengineering, Milan, Italy) with infrared cameras with a sampling rate of 120 Hz, as previously described [12, 19, 20, 21]. Reflective markers were placed on the upper limbs and trunk [19], and three‐dimensional data were acquired in a quiet room, with patients seated comfortably and instructed to perform standardized motor tasks.

Postural tremor was recorded with (i) arms outstretched in front of the chest and (ii) arms flexed in a wing‐beating posture, with three 45‐s trials per condition. Rest tremor was evaluated in three 45‐s recordings with the arms fully relaxed on a table. Kinetic tremor was assessed through three 15‐s recordings of repetitive arm movements. Postural and rest tremor signals were band‐pass filtered at 3–12 Hz, and the power spectrum computed using Welch's method. Tremor frequency was defined as the peak with a half‐power bandwidth < 2 Hz [22], and amplitude as the root mean square (RMS) of the acceleration vector (m/s^2^) within ±1 Hz of the individual peak, averaged across repetitions. For kinetic tremor, the curvature index was computed as the ratio between the actual trajectory length and the straight‐line distance during finger‐to‐goal movements, with higher values reflecting more severe tremor. Repetitive index finger–thumb tapping was recorded in three 15‐s trials per side. Amplitude and velocity were extracted for each tap, and linear regression across consecutive taps was applied to compute amplitude and velocity slopes, capturing sequence effect. For all parameters, values from both sides were averaged; in cases of unilateral tremor, only the affected side was considered.

### 2.4 Statistical Analysis

Continuous variables were summarized using medians and interquartile ranges or means and standard deviations, and categorical variables as counts and percentages. Given the limited sample size, non‐parametric tests were used. Baseline group comparisons employed Mann–Whitney U and Fisher's exact tests, while longitudinal changes were assessed with Friedman's test and Wilcoxon signed‐rank tests for post hoc comparisons. Cochran's Q and McNemar's tests were applied for binary variables.

Delta values were calculated as differences between time points. Associations between continuous variables were assessed using Spearman's correlations, and dichotomous variables were compared using Mann–Whitney U tests. LMMs were fitted for each clinical and kinematic outcome, with TIME (T0, T1, T2) as a fixed effect and random intercepts for subjects; each covariate and its interaction with TIME were modeled separately. Significance was set at *p* < 0.05. False Discovery Rate and Bonferroni correction were applied for multiple comparisons. Analyses were performed using IBM SPSS Statistics 26.

## 3 Results

### 3.1 Baseline Characteristics

Demographic, clinical, and kinematic data are shown in Table 1. Ten patients (45.45%) exhibited tremor involving body regions other than the upper limbs, including voice tremor (*n* = 7), head (*n* = 5), tongue/face (*n* = 2), and lower limbs (*n* = 1), with some overlap. At baseline, females showed a higher number of affected body parts than males (2.20 ± 0.79 vs. 1.25 ± 0.62; Mann–Whitney U = 98.0, *p* = 0.02), which did not survive correction for multiple comparisons. Regarding kinematic data, postural tremor amplitude positively correlated with FTM‐TRS total score and Section A (both ρ = 0.72, *p* < 0.01). Kinematic analysis detected rest tremor in 19 out of 22 patients (86.4%). A positive correlation was found between postural and rest tremor amplitude (*ρ* = 0.44, *p* = 0.03, one‐tailed).

**TABLE 1:** Demographic, clinical, and kinematic measures at baseline and follow‐ups.

	T0	T1	T2	*p*
Sex	12 M (54.55%)	—	—	—
Age (years)	66.05 ± 10.13	69.18 ± 9.91	72.59 ± 10.07	—
Age of onset (years)	53.4 ± 17.77	—	—	—
Tremor duration (years)	12.64 ± 15.16	15.73 ± 15.25	19.14 ± 15.55	
Family history	15 (68.18%)	—	—	—
No. of body segments	1.68 ± 0.84	2.91 ± 1.38	2.86 ± 1.17	< **0.01**
FTM‐TRS total score	21.73 ± 11.58	30.41 ± 14.01	33.46 ± 18.22	< **0.01**
Section A	7.41 ± 4.07	9.64 ± 4.88	10.86 ± 6.27	< **0.01**
Rest tremor	0.27 ± 0.55	0.66 ± 0.64	0.55 ± 0.69	**0.01**
Postural tremor	1.34 ± 0.71	1.32 ± 0.63	1.48 ± 0.84	0.37
Kinetic tremor	1.43 ± 0.56	1.23 ± 0.82	1.36 ± 0.76	0.79
Section B	9.32 ± 5.61	13.05 ± 6.52	14.41 ± 8.57	< **0.01**
Section C	5.00 ± 3.52	7.73 ± 4.94	8.18 ± 5.06	< **0.01**
MDS‐UPDRS III	6.64 ± 4.15	12.82 ± 10.18	14.91 ± 11.39	< **0.01**
MoCA	25.50 ± 2.92	24.00 ± 3.64	24.73 ± 4.80	**0.01**
No. Soft signs	1.09 ± 0.87	1.82 ± 1.30	2.32 ± 1.21	
Rest tremor	5 (22.73%)	10 (45.46%)	12 (54.55%)	**0.04**
Subtle bradykinesia	4 (18.18%)	7 (31.82%)	12 (54.55%)	**0.01**
Questionable dystonia	5 (22.73%)	5 (22.73%)	11 (0.5%)	**0.01**
Impaired tandem gait	2 (9.09%)	6 (27.27%)	7 (31.82%)	0.12
MCI/dementia	8 (36.36%)	12 (54.55%)	9 (40.91%)	0.24
HAM‐A	8.05 ± 7.09	6.64 ± 5.31	7.55 ± 6.70	0.95
HAM‐D	7.41 ± 6.56	5.46 ± 3.90	7.91 ± 5.85	0.57
PT amp (m/s^2^ RMS)	0.22 ± 0.27	0.22 ± 0.34	0.20 ± 0.24	0.25
PT freq (Hz)	5.60 ± 0.77	5.50 ± 0.87	5.18 ± 0.86	< **0.01**
KT amp (CI)	1.06 ± 0.04	1.07 ± 0.04	1.08 ± 0.07	**0.02**
RT amp (m/s^2^ RMS)	0.05 ± 0.05	0.06 ± 0.06	0.07 ± 0.12	0.73
RT freq (Hz)	5.87 ± 0.71	6.14 ± 1.55	5.65 ± 0.88	0.94
FT amp (deg)	49.62 ± 9.50	40.29 ± 11.72	49.01 ± 7.39	0.06
FT vel (deg/s)	1002.12 ± 278.94	926.12 ± 330.99	1003.52 ± 251.31	0.06
FT sl‐amp (deg/mov)	−0.12 ± 0.18	−0.13 ± 0.17	−0.18 ± 0.21	0.83
FT sl‐vel (deg/s/mov)	−6.48 ± 4.43	−5.03 ± 5.76	−6.82 ± 4.23	0.28

*Note:* Data are presented as mean (SD) for descriptive purposes. The median age at onset was 58 years (IQR: 50–65). Five patients had early disease onset (< 45 years), eight in middle adulthood (46–60 years), and nine in late adulthood (> 60 years). The median tremor duration at baseline was 6 years (IQR: 2–19). Median MDS‐UPDRS part III score was 5 (IQR: 3–9), largely reflecting postural and kinetic tremor items, with minimal contribution from bradykinesia‐related scores. Median MoCA score was 26 (IQR: 24–28), median baseline HAM‐A score 4.5 (IQR: 3–12), and median baseline HAM‐D score 7.5 (IQR: 2–11). Slope values are presented as negative numbers to reflect the direction of change, with negative values indicating a decrement in amplitude or velocity over the tapping sequence. Significant results (*p* < 0.05) are reported in bold.

Abbreviations: CI, Curvature Index; deg, degrees; FT, finger tapping; FTM‐TRS, Fahn‐Tolosa‐Marin Tremor Rating Scale; HAM‐A, Hamilton Anxiety Rating Scale; HAM‐D, Hamilton Depression Rating Scale; KT, kinetic tremor; M, males; MDS‐UPDRS III, Movement Disorder Society–sponsored revision of the Unified Parkinson's Disease Rating Scale Part III; MoCA, Montreal Cognitive Assessment; mov, movement; PT, postural tremor; RMS, root mean square; RT, rest tremor; s, seconds; sl‐amp, amplitude slope; sl‐vel, velocity slope.

At baseline, 16 patients (72.73%) were classified as ET‐plus. Rest tremor and questionable dystonia were observed in 5 patients each, subtle bradykinesia in 4, and impaired tandem gait in 2, occurring in various combinations. Notably, only one patient exhibited rest tremor and subtle bradykinesia (namely movement slowness) in combination, with no other features suggestive of parkinsonism [19]. Eight patients (36.36%) were classified as having MCI, and only one patient met criteria for dementia at baseline. MoCA score negatively correlated with age (*ρ* = −0.64, *p* < 0.01).

### 3.2 Longitudinal Changes

**FIGURE 1:**
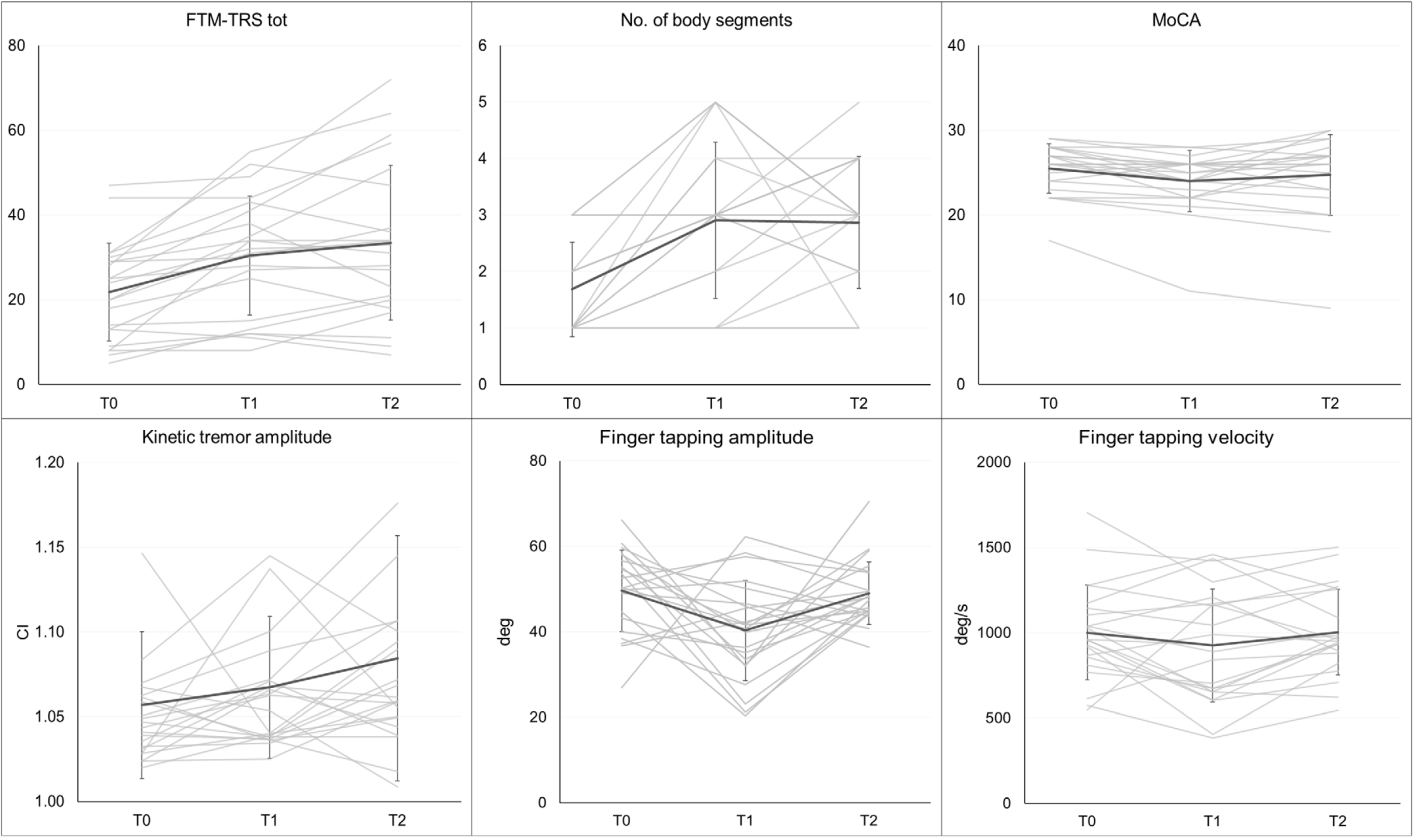
Longitudinal changes of clinical and kinematic measures. Spaghetti plots showing individual trajectories (thin gray lines) and group means with standard deviations (black lines with error bars) across baseline (T0), first follow‐up (T1), and second follow‐up (T2). Clinical measures (top row) include FTM‐TRS total score, number of body segments affected, and MoCA total score. Kinematic measures (bottom row) include kinetic tremor severity (curvature index, CI), finger tapping amplitude expressed in degrees (deg), and finger tapping velocity expressed in degrees/s (deg/s).

Clinical tremor severity worsened over time (Table 1; Figure 1). All FTM‐TRS subsections varied significantly across time points, though post hoc comparisons revealed that only Section B changed between T0 and both follow‐up evaluations, while Sections A and C changed only from T0 to T2. When analyzing the mean scores of rest, postural, and kinetic tremor across both sides separately, only rest tremor showed a significant increase at T1 (Table 1). Similarly, MDS‐UPDRS part III scores increased significantly, with post hoc analysis confirming a worsening between T0 and both T1 and T2. Kinematic analysis showed a significant increase in kinetic tremor severity from T0 to T2 (Table 1; Figure 1), whereas no longitudinal changes were observed in postural or rest tremor amplitude. Tremor anatomical distribution also worsened over time (Table 1).

**FIGURE 2:**
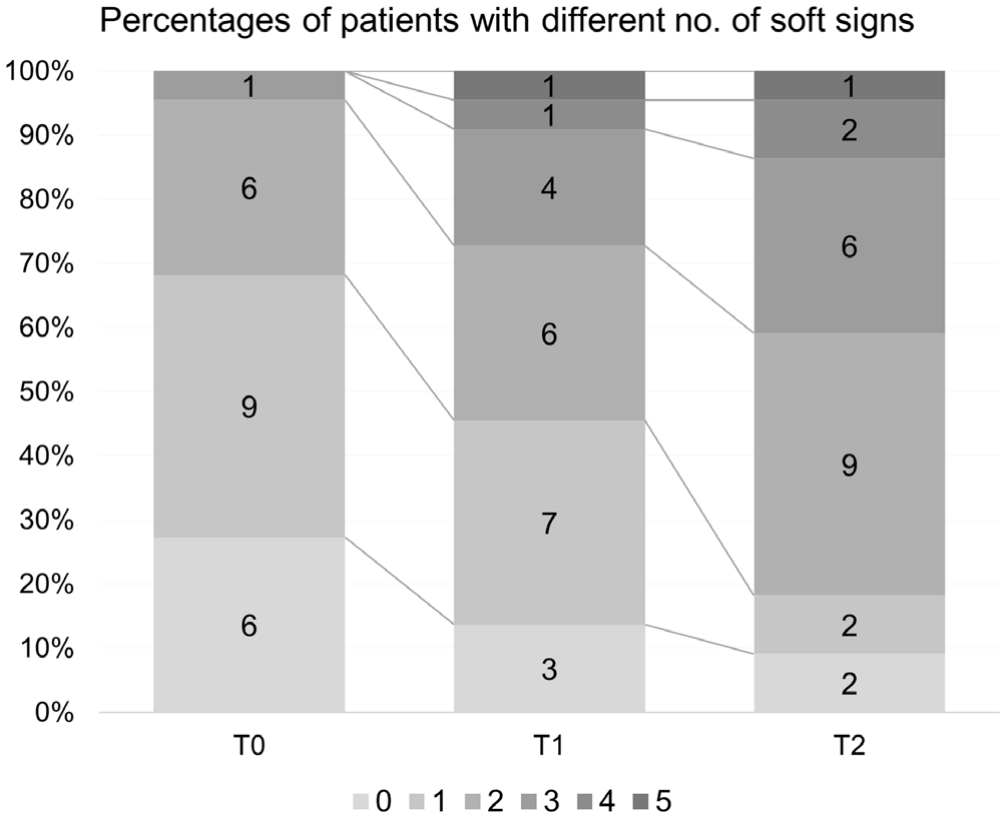
Longitudinal distribution of soft signs. Stacked bar plots showing the percentage of patients with 0 to 5 soft signs at baseline (T0), first follow‐up (T1), and second follow‐up (T2). Numbers within bars indicate the absolute count of patients in each category.

Rest tremor, subtle bradykinesia, and questionable dystonia showed significant overall changes in prevalence over time (Table SS3; Figure 2). After Bonferroni correction, a trend‐level increase was observed at T2 for bradykinesia and questionable dystonia. At T2 the number of patients concurrently exhibiting rest tremor and bradykinesia increased to 6 (27.27%). None showed a clear parkinsonian syndrome or additional signs suggestive of PD [23]. In three of these cases, DaT‐SPECT was performed for diagnostic clarification at the clinician's discretion [3, 24] and was negative. Kinematic data showed a trend‐level change at T1 in both finger tapping amplitude and velocity (Figure 1). MoCA scores showed a milder decline [χ^2^(2) = 8.62, *p* = 0.01], limited to a reduction at T1 (Figure 1). No new dementia diagnoses were made during follow‐up.

Full test statistics are reported in Table SS2.

### 3.3 Correlation and Linear Mixed‐Effects Model Analyses

**FIGURE 3:**
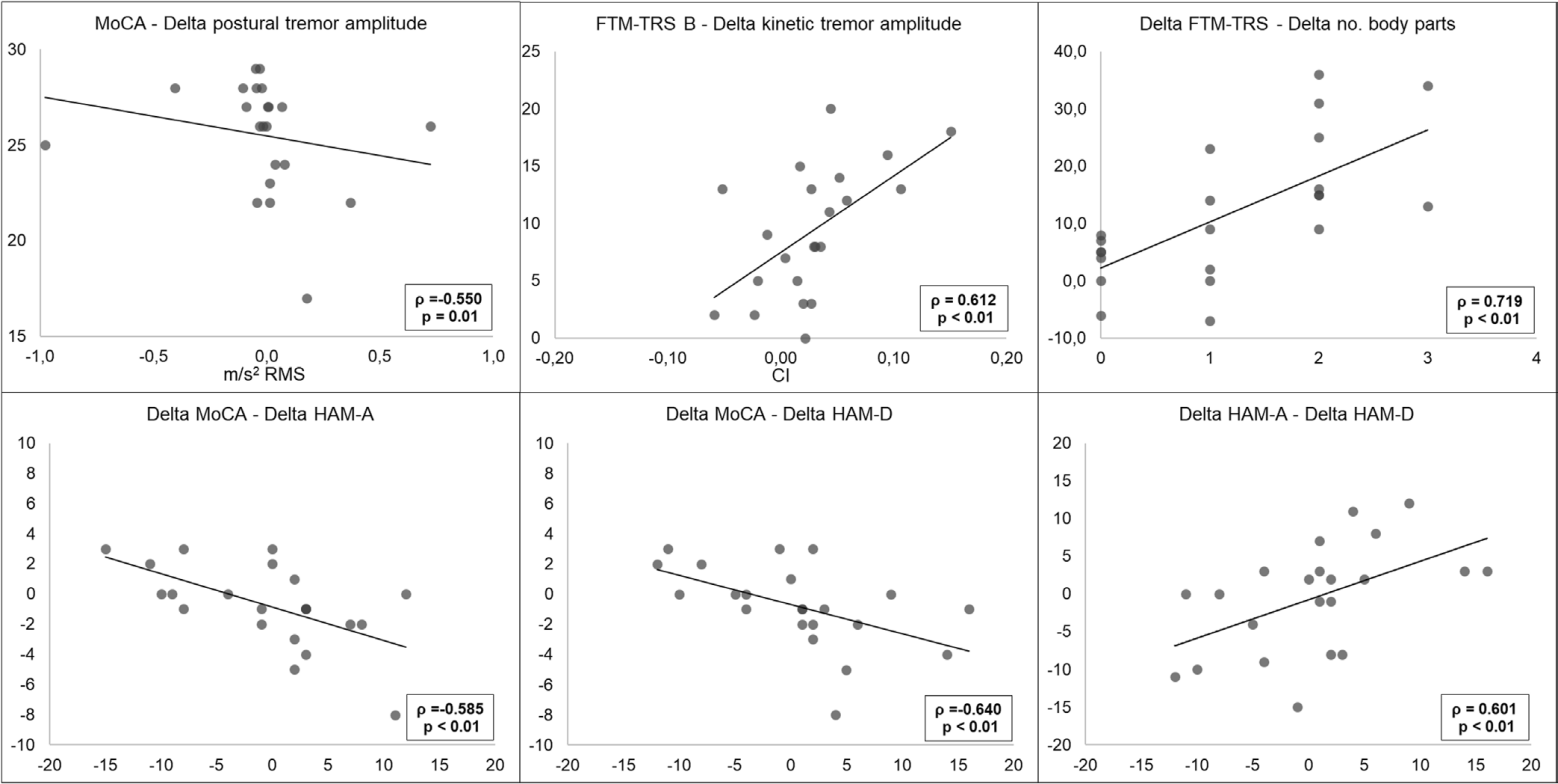
Correlations of clinical and kinematic changes with baseline values and with other longitudinal changes. Scatterplots illustrate significant correlations. Top row: Baseline MoCA score and change in postural tremor amplitude (m/s^2^ RMS); baseline FTM‐TRS Section B and change in kinetic tremor severity (curvature index, CI); changes in FTM‐TRS total score and number of affected body parts. Bottom row: Associations between changes in MoCA score and anxiety (HAM‐A), depression (HAM‐D), and between changes in HAM‐A and HAM‐D scores. Black lines represent linear fit.

No significant correlations were found between delta changes in clinical scores and demographic variables or baseline motor and cognitive scores (all *p* > 0.05). Regarding kinematic parameters, a negative correlation was observed between baseline MoCA scores and postural tremor worsening at T2 (*ρ* = −0.55, *p* = 0.01). Furthermore, higher baseline FTM‐TRS Section B scores were associated with greater worsening of kinetic tremor severity at T2 (*ρ* = 0.61, *p* < 0.01). When analyzing correlations between total delta values of clinical and kinematic parameters, a strong positive association was found between changes in FTM‐TRS total score and changes in the number of body parts involved (*ρ* = 0.72, *p* < 0.01). Greater MoCA decline was associated with greater worsening in anxiety (*ρ* = −0.59, *p* < 0.01) and depression (*ρ* = −0.640, *p* < 0.01) scores (Figure 3).

LMMs confirmed a significant effect of time on the number of body parts affected by tremor, indicating an increase from baseline to follow‐up in models adjusted for sex, tremor duration, baseline number of soft signs, and HAM‐A and HAM‐D scores. In contrast, the effect of time was not significant when age, age of onset, and baseline MoCA score were included as covariates. Similarly, most models showed a significant effect of time on FTM‐TRS total score, indicating clinical worsening across follow‐up. However, this effect was no longer significant when age or baseline MoCA scores were included as covariates. A significant effect of time on the number of soft signs was observed in the model adjusted for sex, indicating a progressive increase over time. Age showed a strong independent effect on the number of soft signs, with older participants presenting a higher soft sign burden regardless of time. The number of body parts at T0 also showed an independent effect, suggesting that individuals with greater tremor spread at baseline also exhibited more soft signs.

Greater baseline FTM‐TRS score was associated with higher postural, kinetic, and rest tremor amplitude objectively assessed across time points. Regardless of time, longer tremor duration, lower MoCA, and a greater number of soft signs were independently associated with higher kinetic and rest tremor severity.

No significant interactions between time and the other covariates were observed for any outcome, indicating that baseline variables did not modify longitudinal trajectories. LMMs full estimates and post hoc contrasts for time are provided in Table SS4.

## 4 Discussion

In this prospective cohort we combined clinical ratings and objective kinematic measures to assess longitudinal changes of motor and non‐motor symptoms in ET over an almost seven‐year follow‐up. Overall, our findings showed that ET progression is heterogeneous within this longitudinally followed cohort. Extended follow‐up confirmed that tremor severity continued to worsen over time, with kinematic data showing an increase in kinetic tremor severity, whereas postural and rest tremor amplitudes remained stable. Clinically, tremor spread anatomically, with soft signs becoming increasingly frequent. Cognition showed only a modest early decline, partly associated with affective symptoms and age. Higher baseline clinical tremor severity was associated with greater worsening of kinetic tremor, and lower baseline MoCA scores were associated with postural tremor worsening. Age was closely associated with disease progression and appeared to play a central role in the evolution of disease burden.

### 4.1 Tremor Progression

In line with our previous study [12], the longer follow‐up observations confirm that ET is a progressive disorder, with clinical worsening mainly reflected in tremor severity and anatomical spread. This may reflect the progressive activity of the tremor‐generating network [25], progressive neurodegeneration [26, 27, 28], or both. Kinematic data showed a selective increase in kinetic tremor severity, whereas postural and rest tremor amplitudes remained largely stable. Notably, in our previous shorter‐term longitudinal study, kinetic tremor did not show significant progression, suggesting that this selective worsening becomes detectable only over longer observation periods and when assessed with objective kinematic measures. Although the effect of time was not confirmed by regression analyses, likely reflecting substantial inter‐individual heterogeneity in longitudinal trajectories, longer tremor duration was nonetheless associated with greater tremor severity. Recent evidence suggests a differential response of postural and kinetic tremor to deep brain stimulation, with the latter showing less suppression, possibly due to oscillations of higher intensity or the recruitment of partially distinct cerebellar–thalamo–cortical regions [29]. The selective worsening of the kinetic component observed here may therefore indicate both a specific amplification of this oscillatory activity over time and a divergence from the mechanisms sustaining postural tremor. This finding is clinically relevant, as kinetic tremor is the most disabling feature of ET and closely correlates with functional impairment [30]. Furthermore, kinetic tremor can include an intention tremor component [31], which has recently been shown to worsen progressively over time [11]. These findings may therefore reflect progression of the underlying cerebellar degeneration [26, 27, 28] and highlight the need for further longitudinal studies integrating clinical, neuroimaging, and neurophysiological measures to better characterize cerebellar involvement in ET [32].

The apparent discrepancy between clinical and kinematic findings reflects the different dimensions captured by these approaches, as clinical scales encompass multiple body regions and functional impact, whereas kinematic assessment was restricted to upper limb tremor. Notably, postural tremor subscores did not change over time, in line with stable kinematic findings, while worsening of kinetic tremor was likely more sensitively captured by objective measures.

### 4.2 Soft Signs Progression

Consistent with previous observations [12, 14], our data confirm a high prevalence of ET‐plus diagnosis and a progressive increase in soft signs over time. This extended follow‐up further showed that, irrespective of time, a greater number of body parts involved at baseline was associated with more soft signs, and a higher burden of soft signs was associated with more severe kinetic tremor. These findings align with evidence that ET‐plus is part of the ET heterogeneity and represents the more severe phenotypes, possibly due to broader network dysfunction or degeneration involving both cerebellar and extracerebellar pathways [33], although not all evidence points in the same direction [27]. Moreover, the observation that patients with longer tremor duration showed greater rest tremor severity may support the hypothesis that ET‐plus, or at least specific subtypes such as ET‐plus with rest tremor, represents a more advanced stage of the disease. From a pathophysiological perspective, we also confirmed a previously reported positive correlation between rest and postural tremor amplitude [19], supporting the idea of shared mechanisms underlying these motor phenomena. Conversely, bradykinesia showed only an early reduction in amplitude and velocity, diverging from the pattern of tremor progression. This reinforces previous indirect evidence that parkinsonian soft signs in ET may arise from distinct underlying pathophysiological mechanisms [19].

Importantly, kinematic analysis detected rest tremor in a much larger proportion of patients compared to clinical examination, underscoring the higher sensitivity of laboratory‐based methods in identifying subclinical tremor features [34]. However, the intermittent nature of rest tremor, together with differences in measurement properties, may limit the ability of short‐duration kinematic recordings to capture longitudinal changes consistently. Clinical ratings provide semi‐quantitative evaluations over longer observation periods, whereas kinematic measures rely on continuous signals acquired over shorter time windows; thus, fluctuating increases in rest tremor may be more readily reflected in clinical scores than in kinematic amplitude measures.

Compared to motor manifestations, cognitive and affective domains appeared relatively stable, and only a mild transient decline in MoCA was detected at the first follow‐up, despite the high prevalence of MCI confirmed in our cohort [35, 36, 37]. This contrasts with part of the literature reporting progressive cognitive deterioration in ET [35, 36], but may be explained by the use of screening tools rather than a neuropsychological battery, as well as by our relatively small sample size.

An intriguing finding was the correlation between changes in MoCA and changes in anxiety and depression scores, in the absence of a direct cross‐sectional association. This suggests that cognitive and affective symptoms may represent partly independent dimensions that nonetheless share common underlying neurodegenerative mechanisms [37, 38] or that progressive cognitive decline may have contributed to affective symptoms, although causality cannot be inferred from the present data. Regarding the influence of non‐motor symptoms on motor performance, lower baseline MoCA scores were associated with greater kinetic and rest tremor severity, supporting the hypothesis of shared pathophysiological substrates between cognitive and motor dysfunction in ET.

### 4.3 Trajectories and Determinants of Disease Evolution

Our cohort showed heterogeneous longitudinal trajectories across both motor and non‐motor domains, confirming that ET progression is not uniform. This variability likely reflects the partly non‐linear course of the disease [10, 11] and the known instability of soft signs over time [13], contributing to its clinical heterogeneity not only in phenotype but also in temporal evolution.

At baseline, female sex was associated with a wider distribution of tremor across body parts. Furthermore, adjusting for sex allowed the effect of time on the number of soft signs to emerge, likely by accounting for inter‐individual heterogeneity. Although not influencing the rate of change, sex therefore is a relevant determinant of tremor expression and heterogeneity [12].

Age emerged as a key determinant of disease burden across multiple domains. Consistent with previous evidence [33, 39, 40], older participants exhibited a greater number of soft signs, independently of time, indicating that age acted as a cross‐sectional correlate of disease severity [41]. Similarly, the longitudinal effects of time on FTM‐TRS and number of body parts affected disappeared when age was included as a covariate, suggesting that part of the observed worsening reflects shared variance between disease progression and age‐related changes. These findings highlight that age and disease progression are deeply intertwined in ET [42], both biologically and statistically. Aging may exacerbate the clinical phenotype through neurodegenerative mechanisms, thereby amplifying the expression of motor and non‐motor features without necessarily accelerating their temporal evolution. In the cognitive domain, the negative correlation between MoCA and age probably supports an age‐related contribution to the mild cognitive decline observed. Overall, our results emphasize the need to disentangle physiological aging from disease progression when interpreting longitudinal changes in ET, particularly in cohorts with wide age ranges or long disease duration.

Among clinical measures, higher baseline FTM‐TRS Section B scores were associated with greater worsening of kinetic tremor severity. This section may capture early motor impairment that anticipates a more pronounced progression of kinetic tremor, the component showing the clearest longitudinal change in kinematic analyses. Finally, an association was found between lower baseline MoCA and postural tremor worsening, hinting that early cognitive disorder may be associated with more severe motor symptoms through shared network mechanisms.

### 4.4 Limitations and Confounding Factors

This study has some limitations. The sample size was relatively small, which may have limited statistical power, particularly for soft‐sign analyses. In addition, given the number of outcomes examined, findings regarding baseline associations should be considered exploratory and interpreted with caution. The small number of patients with early‐onset ET precluded subgroup analyses and limits conclusions regarding the impact of age at onset on longitudinal trajectories. Cognitive assessment relied on a screening tool, potentially underestimating subtle longitudinal changes. Finally, the observational design does not allow full disentanglement of disease‐related progression from age‐related changes.

In addition, several factors may have acted as confounders. Although assessments were conducted under standardized off‐therapy conditions, long‐term treatment exposure and withdrawal effects may still have influenced motor performance over time. Soft neurological signs were clinically defined, and some degree of inter‐rater variability cannot be excluded. Finally, attrition over time may have introduced selection bias. In particular, patients who developed an alternative diagnosis were excluded to preserve diagnostic consistency. As a result, the present cohort represents a selected subset of patients with stable ET who were able to complete repeated kinematic evaluations, potentially underrepresenting more complex or evolving phenotypes.

## 5 Conclusions

This long‐term prospective study supports that ET is a progressive but heterogeneous disorder, with clinical worsening mainly driven by kinetic tremor, anatomical spread, and increase in soft signs. Age influenced disease burden, highlighting the need to disentangle age‐related effects from true progression. Objective kinematic analysis, in combination with clinical assessment, provides objective characterization of tremor and allows detection of subtle changes not fully captured by clinical rating scales. Future longitudinal studies integrating quantitative motor, cognitive, and neurophysiological measures along with biomarkers are warranted to clarify the interplay between aging, soft‐sign accumulation, and network‐level mechanisms driving progression in ET.

## Author Contributions

**Davide Costa:** investigation, data curation. **Adriana Martini:** investigation, data curation. **Sara Cirinei:** investigation, data curation. **Luca Angelini:** conceptualization, data curation, investigation, formal analysis, writing – original draft. **Daniele Birreci:** data curation, investigation. **Martina De Riggi:** investigation, data curation. **Simone Aloisio:** investigation, data curation. **Anna Sofia Grandolfo:** investigation, data curation. **Rick C. G. Helmich:** writing – review and editing. **Giulia Paparella:** investigation, data curation. **Matteo Bologna:** conceptualization, data curation, investigation, formal analysis, writing – original draft.

## Funding

This work was supported by the Italian Ministry of Health (Current Research 2026).

## Conflicts of Interest

The authors declare no conflicts of interest.

## Supporting information


**Table S1:** Baseline clinical characteristics of patients with and without longitudinal follow‐up.

**Table S2:** Longitudinal changes in continuous clinical and kinematic variables.

**Table S3:** Longitudinal changes in prevalence of motor and cognitive soft signs.

**Table S4:** Results of linear mixed‐effects models.

## Data Availability

0The data that support the findings of this study are available on request from the corresponding author. The data are not publicly available due to privacy or ethical restrictions.
