# Supplementary material for: Longitudinal Trajectories in Essential Tremor: Evidence From A Seven‐Year Follow‐Up of Motor and Non‐Motor Symptoms
Source: Eur J Neurol. 2026 Jun 1;33(6):e70646. doi: 10.1111/ene.70646 (PMC13239973; doi:10.1111/ene.70646)
Supplement: Supplementary file 1 — Table S1: Baseline clinical characteristics of patients with and without longitudinal follow‐up. [file ENE-33-e70646-s004.docx]

**Supplementary Table 1. Baseline clinical characteristics of patients with and without longitudinal follow-up**

|  | **Dropout (n=15)** | **Follow-up (n=22)** | **p** |
| --- | --- | --- | --- |
| Sex | 9M (60.00%) | 13M (59.09%) | 0.96 |
| Age (years) | 70 (66-72) | 67 (61-72) | 0.71^a^ |
| Age of onset (years) | 56 (45-62) | 58 (50-65) | 0.56^a^ |
| Tremor duration (years) | 10 (7-14) | 10 (5-28) | 0.89^a^ |
| Family history | 7Y (46.67%) | 16Y (72.73%) | 0.11 |
| No. of body segments | 2 (1-2) | 1 (1-2) | 0.80^a^ |
| FTM-TRS total score | 20 (9-25) | 22.5 (13-30) | 0.33^b^ |
| - Section A | 7 (5-8) | 7 (4-10) | 0.45^b^ |
| - Section B | 7 (4-11) | 8.5 (5-14) | 0.35^b^ |
| - Section C | 5 (1-6) | 4.5 (2-7) | 0.45^b^ |
| MDS-UPDRS III | 6 (5-8) | 5 (3-9) | 0.91^a^ |
| MoCA | 25 (24-26) | 26 (24-28) | 0.12^a^ |
| No. soft signs | 1 (1-2) | 1 (0-2) | 0.55^a^ |
| HAM-A | 10 (0-15) | 4 (2-14) | 0.98^a^ |
| HAM-D | 8 (0-11) | 7 (2-12) | 0.86^a^ |

M: males; Y: yes; FTM-TRS: Fahn-Tolosa-Marin Tremor Rating Scale; MDS-UPDRS III: Movement Disorder Society–sponsored revision of the Unified Parkinson’s Disease Rating Scale, Part III; MoCA: Montreal Cognitive Assessment; HAM-A: Hamilton Anxiety Rating Scale; HAM-D: Hamilton Depression Rating Scale. Baseline demographic and clinical characteristics are reported separately for patients who lost to follow-up (Dropout) and those with longitudinal follow-up (Follow-up). Numerical variables are reported as median (interquartile range), and categorical variables as number (percentage). ^a^: Mann–Whitney U test; ^b^: unpaired Student’s t‑test.
