# Supplementary material for: Longitudinal Trajectories in Essential Tremor: Evidence From A Seven‐Year Follow‐Up of Motor and Non‐Motor Symptoms
Source: Eur J Neurol. 2026 Jun 1;33(6):e70646. doi: 10.1111/ene.70646 (PMC13239973; doi:10.1111/ene.70646)
Supplement: Supplementary file 2 — Table S2: Longitudinal changes in continuous clinical and kinematic variables. [file ENE-33-e70646-s003.docx]

**Supplementary Table 2. Longitudinal changes in continuous clinical and kinematic variables**

| **Variable** | **χ²(2)** | **p** | **T0–T1 p_adj** | **T0–T2 p_adj** | **T1–T2 p_adj** |
| --- | --- | --- | --- | --- | --- |
| No. body segments | 19.91 | **<0.01** | **0.01** | **<0.01** | 1.00 |
| FTM-TRS total score | 20.99 | **<0.01** | **<0.01** | **<0.01** | 1.00 |
| **-** Section A | 10.83 | **<0.01** | 0.07 | **0.01** | 1.00 |
| **-** Section B | 12.17 | **<0.01** | **0.02** | **0.01** | 1.00 |
| **-** Section C | 15.18 | **<0.01** | 0.09 | **<0.01** | 1.00 |
| MDS-UPDRS III | 14.34 | **<0.01** | **0.01** | **0.01** | 1.00 |
| MoCA | 8.62 | **0.01** | **0.02** | 0.21 | 1.00 |
| HAM-A | 0.10 | 0.95 | – | – | – |
| HAM-D | 1.13 | 0.57 | – | – | – |
| PT amp | 2.80 | 0.25 | – | – | – |
| PT freq | 12.49 | **<0.01** | 0.14 | **<0.01** | 0.40 |
| KT amp | 7.49 | **0.02** | 0.46 | **0.03** | 0.68 |
| RT amp | 0.64 | 0.73 | – | – | – |
| RT freq | 0.13 | 0.94 | – | – | – |
| FT amp | 5.55 | 0.06 | – | – | – |
| FT vel | 5.73 | 0.06 | – | – | – |
| FT sl-amp | 0.36 | 0.83 | – | – | – |
| FT sl-vel | 2.55 | 0.28 | – | – | – |

FTM-TRS: Fahn-Tolosa-Marin Tremor Rating Scale; MDS-UPDRS III: Movement Disorder Society–sponsored revision of the Unified Parkinson’s Disease Rating Scale, Part III; MoCA: Montreal Cognitive Assessment; HAM-A: Hamilton Anxiety Rating Scale; HAM-D: Hamilton Depression Rating Scale; PT: postural tremor; RT: rest tremor; KT: kinetic tremor; FT: finger tapping; amp: amplitude; freq: frequency; sl-amp: amplitude slope; sl-vel: velocity slope.
p-values refer to Friedman test; post hoc comparisons were performed using Wilcoxon signed-rank tests with Bonferroni correction; adjusted p-values (p_adj) are shown. Significant results (p < 0.05) are reported in bold.
