# Supplementary material for: Longitudinal Trajectories in Essential Tremor: Evidence From A Seven‐Year Follow‐Up of Motor and Non‐Motor Symptoms
Source: Eur J Neurol. 2026 Jun 1;33(6):e70646. doi: 10.1111/ene.70646 (PMC13239973; doi:10.1111/ene.70646)
Supplement: Supplementary file 3 — Table S3: Longitudinal changes in prevalence of motor and cognitive soft signs. [file ENE-33-e70646-s002.docx]

**Supplementary Table 3.** **Longitudinal changes in prevalence of motor and cognitive soft signs**

| **Soft Sign** | **χ² (2)** | **p** | **T0–T1 p_adj** | **T0–T2 p_adj** | **T1–T2 p_adj** |
| --- | --- | --- | --- | --- | --- |
| Rest tremor | 6.50 | **0.039** | 0.375 | 0.195 | 1.000 |
| Bradykinesia | 9.80 | **0.007** | 0.750 | 0.063 | 0.375 |
| Questionable dystonia | 9.00 | **0.011** | 1.000 | 0.210 | 0.093 |
| Impaired tandem gait | 4.20 | 0.122 | 0.375 | 0.375 | 1.000 |
| MCI | 2.89 | 0.236 | 0.867 | 1.000 | 1.000 |

MCI: mild cognitive impairment. p-values refer to Cochran’s Q test; post hoc comparisons were performed using McNemar’s test with Bonferroni correction; adjusted p values (p_adj) are shown. Significant p values are shown in bold.
