# Supplementary material for: Longitudinal Trajectories in Essential Tremor: Evidence From A Seven‐Year Follow‐Up of Motor and Non‐Motor Symptoms
Source: Eur J Neurol. 2026 Jun 1;33(6):e70646. doi: 10.1111/ene.70646 (PMC13239973; doi:10.1111/ene.70646)
Supplement: Supplementary file 4 — Table S4: Results of linear mixed‐effects models. [file ENE-33-e70646-s001.docx]

**Supplementary Table 4. Results of linear mixed-effects models**

| **Outcome** | **Covariate** | **Fixed effect** | **F(df)** | **p** | **Post hoc TIME** |
| --- | --- | --- | --- | --- | --- |
| No. BODY PARTS | SEX | TIME | F(2,40)=12.414 | **<0.001** | T0 < T1; T0 < T2 |
|  |  | SEX | F(1,20)=8.448 | 0.009 | – |
|  |  | TIME×SEX | F(2,40)=0.011 | 0.989 | – |
|  | AGE | TIME | F(2,40)=1.597 | 0.215 | – |
|  |  | AGE | F(1,20)=0.169 | 0.686 | – |
|  |  | TIME×AGE | F(2,40)=1.029 | 0.366 | – |
|  | AGE ONSET | TIME | F(2,40)=3.443 | 0.042 | – |
|  |  | AGE ONSET | F(1,20)=0.002 | 0.968 | – |
|  |  | TIME×AGE ONSET | F(2,40)=1.065 | 0.354 | – |
|  | TREMOR DURATION | TIME | F(2,40)=5.856 | **0.006** | T0 < T1; T0 < T2 |
|  |  | TREMOR DURATION | F(1,20)=0.104 | 0.751 | – |
|  |  | TIME×TREMOR DURATION | F(2,40)=0.345 | 0.711 | – |
|  | MOCA T0 | TIME | F(2,40)=0.389 | 0.681 | – |
|  |  | MOCA T0 | F(1,20)=0.171 | 0.683 | – |
|  |  | TIME×MOCA T0 | F(2,40)=0.505 | 0.607 | – |
|  | No. SS T0 | TIME | F(2,40)=7.629 | **0.002** | T0 < T1; T0 < T2 |
|  |  | No. SS T0 | F(1,20)=1.586 | 0.222 | – |
|  |  | TIME×No. SS T0 | F(2,40)=1.027 | 0.367 | – |
|  | HAM-A T0 | TIME | F(2,40)=5.021 | **0.011** | T0 < T1; T0 < T2 |
|  |  | HAM-A T0 | F(1,20)=0.009 | 0.924 | – |
|  |  | TIME×HAM-A T0 | F(2,40)=0.130 | 0.879 | – |
|  | HAM-D T0 | TIME | F(2,40)=5.649 | **0.007** | T0 < T1; T0 < T2 |
|  |  | HAM-D T0 | F(1,20)=0.517 | 0.481 | – |
|  |  | TIME×HAMD T0 | F(2,40)=0.326 | 0.723 | – |
|  |  |  |  |  |  |
|  |  |  |  |  |  |
|  |  |  |  |  |  |
|  |  |  |  |  |  |
| FTM-TRS TOTAL | SEX | TIME | F(2,40)=15.581 | **<0.001** | T0 < T1; T0 < T2 |
|  |  | SEX | F(1,20)=0.000 | 0.999 | – |
|  |  | TIME×SEX | F(2,40)=0.578 | 0.566 | – |
|  | AGE | TIME | F(2,40)=0.745 | 0.481 | – |
|  |  | AGE | F(1,20)=3.846 | 0.064 | – |
|  |  | TIME×AGE | F(2,40)=2.191 | 0.125 | – |
|  | AGE ONSET | TIME | F(2,40)=3.659 | **0.035** | T0 < T1; T0 < T2 |
|  |  | AGE ONSET | F(1,20)=0.074 | 0.788 | – |
|  |  | TIME ×AGE ONSET | F(2,40)=0.544 | 0.584 | – |
|  | TREMOR DURATION (YEARS) | TIME | F(2,40)=4.200 | **0.022** | T0 < T1; T0 < T2 |
|  |  | TREMOR DURATION | F(1,20)=2.602 | 0.122 | – |
|  |  | TIME×TREMOR DURATION | F(2,40)=3.520 | 0.039 | – |
|  | MOCA T0 | TIME | F(2,40)=1.345 | 0.272 | – |
|  |  | MOCA T0 | F(1,20)=1.030 | 0.322 | – |
|  |  | TIME×MOCA T0 | F(2,40)=0.564 | 0.573 | – |
|  | No. SS T0 | TIME | F(2,40)=6.264 | **0.004** | T0 < T1; T0 < T2 |
|  |  | No. SS T0 | F(1,20)=4.225 | 0.053 | – |
|  |  | TIME×No. SS T0 | F(2,40)=0.036 | 0.965 | – |
|  | HAM-A T0 | TIME | F(2,40)=10.268 | **<0.001** | T0 < T1; T0 < T2 |
|  |  | HAM-A T0 | F(1,20)=0.084 | 0.775 | – |
|  |  | TIME×HAM-A T0 | F(2,40)=0.599 | 0.554 | – |
|  | HAM-D T0 | TIME | F(2,40)=8.126 | **0.001** | T0 < T1; T0 < T2 |
|  |  | HAM-D T0 | F(1,20)=0.063 | 0.805 | – |
|  |  | TIME×HAM-D T0 | F(2,40)=0.186 | 0.831 | – |
|  |  |  |  |  |  |
|  |  |  |  |  |  |
|  |  |  |  |  |  |
|  |  |  |  |  |  |
|  |  |  |  |  |  |
| MOCA | SEX | TIME | F(2,40)=4.34 | 0.020 | – |
|  |  | SEX | F(1,20)=0.03 | 0.871 | – |
|  |  | TIME×SEX | F(2,40)=0.14 | 0.868 | – |
|  | AGE | TIME | F(2,40)=1.50 | 0.235 | – |
|  |  | AGE | F(1,20)=6.87 | 0.016 | – |
|  |  | TIME×AGE | F(2,40)=1.42 | 0.253 | – |
|  | AGE ONSET | TIME | F(2,40)=1.24 | 0.302 | – |
|  |  | AGE ONSET | F(1,20)=0.92 | 0.348 | – |
|  |  | TIME×AGE ONSET | F(2,40)=0.54 | 0.586 | – |
|  | TREMOR DURATION | TIME | F(2,40)=1.86 | 0.169 | – |
|  |  | TREMOR DURATION | F(1,20)=0.17 | 0.685 | – |
|  |  | TIME×TREMOR DURATION | F(2,40)=0.09 | 0.910 | – |
|  | No. SS T0 | TIME | F(2,40)=1.95 | 0.156 | – |
|  |  | No. SS T0 | F(1,20)=9.82 | **0.005** | – |
|  |  | TIME×No. SS T0 | F(2,40)=1.01 | 0.374 | – |
|  | HAM‑A T0 | TIME | F(2,40)=3.72 | 0.033 | – |
|  |  | HAM‑A T0 | F(1,20)=0.25 | 0.626 | – |
|  |  | TIME×HAM‑A T0 | F(2,40)=0.67 | 0.519 | – |
|  | HAM‑D T0 | TIME | F(2,40)=2.92 | 0.065 | – |
|  |  | HAM‑D T0 | F(1,20)=0.00 | 0.953 | – |
|  |  | TIME×HAM‑D T0 | F(2,40)=0.95 | 0.397 | – |
|  |  |  |  |  |  |
|  |  |  |  |  |  |
|  |  |  |  |  |  |
|  |  |  |  |  |  |
|  |  |  |  |  |  |
|  |  |  |  |  |  |
|  |  |  |  |  |  |
|  |  |  |  |  |  |
| No. SS tot | SEX | TIME | F(2,60)=6.16 | **0.004** | T0<T1, T0<T2 |
|  |  | SEX | F(1,60)=0.00 | 0.954 | – |
|  |  | TIME×SEX | F(2,60)=0.14 | 0.871 | – |
|  | AGE | TIME | F(2,60)=0.06 | 0.940 | – |
|  |  | AGE | F(1,60)=19.39 | **<0.001** | – |
|  |  | TIME×AGE | F(2,60)=0.36 | 0.701 | – |
|  | AGE ONSET | TIME | F(2,60)=0.14 | 0.868 | – |
|  |  | AGE ONSET | F(1,60)=3.61 | 0.062 | – |
|  |  | TIME×AGE ONSET | F(2,60)=0.24 | 0.789 | – |
|  | TREMOR DURATION | TIME | F(2,60)=4.14 | 0.021 | – |
|  |  | TREMOR DURATION | F(1,60)=0.15 | 0.697 | – |
|  |  | TIME×TREMOR DURATION | F(2,60)=0.13 | 0.879 | – |
|  | No. BODY PARTS T0 | TIME | F(2,60)=1.22 | 0.301 | – |
|  |  | No. BODY PARTS T0 | F(1,60)=9.84 | **0.003** | – |
|  |  | TIME× No. BODY PARTS T0 | F(2,60)=0.07 | 0.935 | – |
|  | HAM-A T0 | TIME | F(2,60)=3.94 | 0.025 | – |
|  |  | HAM-A T0 | F(1,60)=1.67 | 0.201 | – |
|  |  | TIME×HAM-A T0 | F(2,60)=0.47 | 0.629 | – |
|  | HAM-D T0 | TIME | F(2,60)=3.93 | 0.025 | – |
|  |  | HAM-D T0 | F(1,60)=0.03 | 0.857 | – |
|  |  | TIME×HAM-D T0 | F(2,60)=0.35 | 0.710 | – |
|  |  |  |  |  |  |
|  |  |  |  |  |  |
|  |  |  |  |  |  |
|  |  |  |  |  |  |
|  |  |  |  |  |  |
|  |  |  |  |  |  |
|  |  |  |  |  |  |
|  |  |  |  |  |  |
| PT AMPLITUDE | SEX | TIME | F(2,58)=0.035 | 0.966 | – |
|  |  | SEX | F(1,58)=0.946 | 0.335 | – |
|  |  | TIME×SEX | F(2,58)=0.623 | 0.540 | – |
|  | AGE | TIME | F(2,58)=0.048 | 0.953 | – |
|  |  | AGE | F(1,58)=2.436 | 0.124 | – |
|  |  | TIME×AGE | F(2,58)=0.043 | 0.958 | – |
|  | AGE ONSET | TIME | F(2,58)=0.151 | 0.860 | – |
|  |  | AGE ONSET | F(1,58)=0.914 | 0.343 | – |
|  |  | TIME×AGE ONSET | F(2,58)=0.180 | 0.835 | – |
|  | TREMOR DURATION | TIME | F(2,58)=0.145 | 0.866 | – |
|  |  | TREMOR DURATION | F(1,58)=0.001 | 0.972 | – |
|  |  | TIME×TREMOR DURATION | F(2,58)=0.187 | 0.830 | – |
|  | FTM-TRS T0 | TIME | F(2,58)=0.405 | 0.669 | – |
|  |  | FTM-TRS T0 | F(1,58)=40.358 | **<0.001** | – |
|  |  | TIME×FTM-TRS T0 | F(2,58)=0.611 | 0.546 | – |
|  | MOCA T0 | TIME | F(2,58)=0.347 | 0.708 | – |
|  |  | MOCA T0 | F(1,58)=0.202 | 0.655 | – |
|  |  | TIME×MOCA T0 | F(2,58)=0.372 | 0.691 | – |
|  | No. SS T0 | TIME | F(2,58)=0.141 | 0.869 | – |
|  |  | No. SS T0 | F(1,58)=3.215 | 0.078 | – |
|  |  | TIME× No. SS T0 | F(2,58)=0.356 | 0.702 | – |
|  | HAM-A T0 | TIME | F(2,58)=0.155 | 0.856 | – |
|  |  | HAM-A T0 | F(1,58)=0.190 | 0.664 | – |
|  |  | TIME×HAM-A T0 | F(2,58)=0.163 | 0.850 | – |
|  | HAM-D T0 | TIME | F(2,58)=0.225 | 0.799 | – |
|  |  | HAM-D T0 | F(1,58)=0.281 | 0.598 | – |
|  |  | TIME×HAM-D T0 | F(2,58)=0.263 | 0.770 | – |
|  |  |  |  |  |  |
|  |  |  |  |  |  |
| KT AMPLITUDE | SEX | TIME | F(2,60)=1.316 | 0.276 | – |
|  |  | SEX | F(1,60)=0.083 | 0.774 | – |
|  |  | TIME×SEX | F(2,60)=0.406 | 0.668 | – |
|  | AGE | TIME | F(2,60)=0.317 | 0.729 | – |
|  |  | AGE | F(1,60)=3.015 | 0.088 | – |
|  |  | TIME×AGE | F(2,60)=0.454 | 0.637 | – |
|  | AGE ONSET | TIME | F(2,60)=0.559 | 0.575 | – |
|  |  | AGE ONSET | F(1,60)=0.904 | 0.346 | – |
|  |  | TIME×AGE ONSET | F(2,60)=0.195 | 0.824 | – |
|  | TREMOR DURATION | TIME | F(2,60)=0.243 | 0.785 | – |
|  |  | TREMOR DURATION | F(1,60)=5.490 | **0.022** | – |
|  |  | TIME×TREMOR DURATION | F(2,60)=0.515 | 0.600 | – |
|  | FTM-TRS T0 | TIME | F(2,60)=1.052 | 0.355 | – |
|  |  | FTM-TRS T0 | F(1,60)=30.887 | **<0.001** | – |
|  |  | TIME×FTM-TRS T0 | F(2,60)=3.187 | 0.048 | – |
|  | MOCA T0 | TIME | F(2,60)=1.000 | 0.374 | – |
|  |  | MOCA T0 | F(1,60)=21.040 | **<0.001** | – |
|  |  | TIME×MOCA T0 | F(2,60)=0.793 | 0.457 | – |
|  | No. SS T0 | TIME | F(2,60)=0.319 | 0.728 | – |
|  |  | No. SS T0 | F(1,60)=23.393 | **<0.001** | – |
|  |  | TIME× No. SS T0 | F(2,60)=0.942 | 0.396 | – |
|  | HAM-A T0 | TIME | F(2,60)=1.523 | 0.226 | – |
|  |  | HAM-A T0 | F(1,60)=1.457 | 0.232 | – |
|  |  | TIME×HAM-A T0 | F(2,60)=0.409 | 0.666 | – |
|  | HAM-D T0 | TIME | F(2,60)=0.952 | 0.392 | – |
|  |  | HAM-D T0 | F(1,60)=3.387 | 0.071 | – |
|  |  | TIME×HAM-D T0 | F(2,60)=0.374 | 0.690 | – |
|  |  |  |  |  |  |
|  |  |  |  |  |  |
| RT AMPLITUDE | SEX | TIME | F(2,51)=0.283 | 0.755 | – |
|  |  | SEX | F(1,51)=2.675 | 0.108 | – |
|  |  | TIME×SEX | F(2,51)=0.713 | 0.495 | – |
|  | AGE | TIME | F(2,51)=0.330 | 0.721 | – |
|  |  | AGE | F(1,51)=1.819 | 0.183 | – |
|  |  | TIME×AGE | F(2,51)=0.444 | 0.644 | – |
|  | AGE ONSET | TIME | F(2,51)=1.298 | 0.282 | – |
|  |  | AGE ONSET | F(1,51)=4.603 | 0.037 | – |
|  |  | TIME×AGE ONSET | F(2,51)=1.173 | 0.318 | – |
|  | TREMOR DURATION | TIME | F(2,51)=0.821 | 0.446 | – |
|  |  | TREMOR DURATION | F(1,51)=14.331 | **<0.001** | – |
|  |  | TIME×TREMOR DURATION | F(2,51)=3.641 | 0.033 | – |
|  | FTM-TRS T0 | TIME | F(2,51)=0.564 | 0.572 | – |
|  |  | FTM-TRS T0 | F(1,51)=14.998 | **<0.001** | – |
|  |  | TIME×FTM-TRS T0 | F(2,51)=1.236 | 0.299 | – |
|  | MOCA T0 | TIME | F(2,51)=1.032 | 0.364 | – |
|  |  | MOCA T0 | F(1,51)=6.532 | **0.014** | – |
|  |  | TIME×MOCA T0 | F(2,51)=0.910 | 0.409 | – |
|  | No. SS T0 | TIME | F(2,51)=0.164 | 0.849 | – |
|  |  | No. SS T0 | F(1,51)=5.941 | **0.018** | – |
|  |  | TIME× No. SS T0 | F(2,51)=0.650 | 0.526 | – |
|  | HAM-A T0 | TIME | F(2,51)=0.033 | 0.968 | – |
|  |  | HAM-A T0 | F(1,51)=0.681 | 0.413 | – |
|  |  | TIME×HAM-A T0 | F(2,51)=0.171 | 0.844 | – |
|  | HAM-D T0 | TIME | F(2,51)=0.026 | 0.975 | – |
|  |  | HAM-D T0 | F(1,51)=1.729 | 0.194 | – |
|  |  | TIME×HAM-D T0 | F(2,51)=0.054 | 0.947 | – |

FTM-TRS: Fahn-Tolosa-Marin Tremor Rating Scale; MoCA: Montreal Cognitive Assessment; HAM-A: Hamilton Anxiety Rating Scale; HAM-D: Hamilton Depression Rating Scale; SS: soft signs; KT: kinetic tremor; PT: postural tremor; RT: rest tremor. Random intercepts for subjects were included. F statistics derive from Type III tests of fixed effects. P-values were adjusted for multiple testing using false discovery rate (FDR) correction (Benjamini–Hochberg), applied separately to TIME effects, covariates, and TIME×covariate interactions. Significant p values are shown in bold. Only significant post hoc pairwise comparisons for the factor TIME are reported.
